# Hormesis Effects of Silver Nanoparticles at Non-Cytotoxic Doses to Human Hepatoma Cells

**DOI:** 10.1371/journal.pone.0102564

**Published:** 2014-07-17

**Authors:** Zhi-Hao Jiao, Ming Li, Yi-Xing Feng, Jia-Chen Shi, Jing Zhang, Bing Shao

**Affiliations:** Beijing Key Laboratory of Diagnostic and Traceability Technologies for Food Poisoning, Beijing Center for Disease Control and Prevention, Beijing, China; Helmholtz Zentrum München, Germany

## Abstract

Silver nanoparticles (AgNPs) have attracted considerable attentions due to their unique properties and diverse applications. Although it has been reported that AgNPs have acute toxic effects on a variety of cultured mammalian cells and animal models, few studies have been conducted to evaluate the associated risk of AgNPs to human health at non-cytotoxic doses. In this paper, HepG2 cells were exposed to 10 nm and 100 nm AgNPs under non-cytotoxic conditions, and cell viability was assessed. At low doses, AgNPs displayed “hormesis” effects by accelerating cell proliferation. Further studies indicated that the activation states of MAPKs were differentially regulated in this process. Specifically, by increasing the expression of downstream genes, p38 MAPK played a central role in non-cytotoxic AgNP-induced hormesis. Moreover, the treatment of HepG2 cells with silver ions (Ag^+^) at the same dose levels induced distinct biological effects, suggesting that different intrinsic properties exist for AgNPs and Ag^+^.

## Introduction

Nanoparticles (NPs), defined as structures with at least one dimension of 100 nanometers or less [1], have been widely utilized due to their unique physical and chemical properties [2]. AgNPs possess antimicrobial properties [3] and have been broadly applied in medical and consumer products, such as disinfectants for medical devices, food packaging and clothing [4], [5], [6]. Additionally, their special optical properties also allow AgNPs to be incorporated into biological and chemical sensors [7], [8].

Despite of the widespread use and increased human and environmental exposure to AgNPs [9], [10], systematic toxicological information is still lacking [11]. *In vivo* bio-distribution and toxicity studies on exposure to AgNPs via inhalation or ingestion in mammalian animal models have revealed that AgNPs may cause toxicity to several target organs, such as the liver, kidney, spleen, brain and lung [12], [13], [14], [15]. Of note, Kim et al. reported significant hepatic changes in alkaline phosphatase activity, cholesterol level and slight liver damage in rats following 28-day oral AgNPs exposure [16]. In a 90-day inhalation exposure study in rats, Sung et al. reported an increase in bile duct hyperplasia and liver inflammation [14]. Hepatotoxicity of AgNPs after 3-day oral exposure in mice was also reported by Cha et al., with lymphocytic infiltration and the expression of genes related to apoptosis and inflammation in the liver [17].

The toxicity of AgNPs has also been investigated in various mammalian cell models. These *in vitro* studies have also shown that AgNPs are able to interfere with cellular functions and cause toxic effects, including DNA damage and apoptosis [18], [19], [20], [21], [22], [23], [24]. The induction of oxidative stress is the most commonly reported mechanism of AgNPs toxicity, which is the consequence of the generation of intracellular reactive oxygen species (ROS) within the cells [25], [26]. ROS and oxidative stress may elicit cellular events including DNA damage and apoptosis [27], [28].

However, in general, most of the existing studies have evaluated the acute toxic effects of AgNPs at relatively high doses, while their potential risk at relatively low doses has not been defined. The purpose of this study was to investigate the potential biological effects of AgNPs at non-cytotoxic doses. We selected two representative AgNPs dispersions with 10 nm and 100 nm particle sizes, respectively. In addition, to distinguish between the direct “particle-specific” effects and the indirect released Ag^+^ induced effects, Ag^+^ was also analyzed in parallel at the same dose levels as AgNPs.

In this study, human hepatoma-derived cell line HepG2 was employed as an *in vitro* model, as liver is a major target organ of AgNPs [11], and HepG2 is the cell line that is most widely used in evaluating the *in vitro* toxicity of AgNPs among all the liver cell lines [25], [29], [30], [31], [32]. First, the cell cytotoxicity and ROS generation caused by AgNPs exposure were examined to define the non-cytotoxic concentration ranges of AgNPs. The cell viability and proliferation were next detected as the basis of cellular responses. Further, the mechanisms by which AgNPs influence these biological processes remain to be explored. Thus, we analyzed the cellular alterations caused by AgNPs exposure at the protein level, focusing on the evolutionally conserved MAPK signaling pathways, which regulate cell growth, differentiation, apoptosis and transformation through intracellular phosphorylation [33], [34]. We detected the expression levels, activation states and downstream triggers of MAPK family members including c-Jun N-terminal kinase (JNK), extracellular signal-regulating kinase (ERK) and p38 protein kinase. Finally, the roles of MAPKs on the AgNP-induced biological effects were determined. These results may provide more evidence on the potential risk of non-cytotoxic AgNPs to human health.

## Materials and Methods

### Chemicals

Two types of AgNPs were purchased from Sigma-Aldrich (St. Louis, MO, USA, catalog No. 730785 & 730777). According to the product information, both of the AgNPs were supplied at a concentration of 0.02 mg/mL dispersed in aqueous buffer, containing 2 mM sodium citrate as a stabilizer to prevent aggregation. Their particle sizes were 10 nm and 100 nm, respectively. Silver nitrate (AgNO_3_) and N-acetyl cysteine were also purchased from Sigma. Ag^+^ stock solution (1000 µg/ml) prepared in 1% HNO_3_ was purchased from National Institute of Metrology (Beijing, China). The MAPK family antibody sampler kit (#9926) and Phospho-MAPK family antibody sampler kit (#9910) were obtained from Cell Signaling Technology, Inc. (Beverly, MA, USA). The anti-β-actin antibody was obtained from Pierce (Rockford, IL, USA). The p38 MAPK inhibitor SB203580 was obtained from Beyotime Institute of Biotechnology (China).

### Characterization of AgNPs

The primary sizes and morphology of AgNPs were examined using a transmission electronic microscope (JEM-1200EX, JEOL Ltd., Japan). After sonicating in a water bath, the AgNPs stock solutions were placed over a copper grid coated with carbon film and air dried overnight for TEM analysis. The images were processed with a particle analysis tool (Nano Measurer, Fudan University, China) to establish size distributions.

The hydrodynamic diameter and zeta potential of AgNPs were characterized by dynamic light scattering (DLS) using a Malvern Zetasizer Nano-ZS (Malvern Instruments Ltd., Worcestershire, UK). Besides characterizing the stock suspensions, they were also diluted 1∶20 in cell culture medium (detailed in the cell culture part), and the measurements were performed after 5 minutes, 1 hour, 24 hours, 48 hours and 72 hours. Each measurement was run in triplicate using automated measurement times.

### Quantification of the AgNPs Stock Solutions

The total Ag contents in the AgNPs stock solutions were measured by an Agilent 8800 ICP-MS Triple Quad Instrument (Agilent Technologies, Japan) with Ag standard solutions prepared by diluting a certified reference material. 3 mL 65% Nitric Acid were added to 100 µL AgNPs stock solutions followed by microwave-assisted digestion (CEM Mars 5, Xpress, Matthews, NC). After digestion, samples were diluted with water to 40 mL for ICP-MS determination.

The amounts of silver ions released from AgNPs stock solutions were measured using an Orion 3 Star pH Benchtop Meter (Thermo Scientific, Beverly, MA) with a silver/sulfide ion-selective electrode. Serial dilutions of AgNO_3_ solution (0, 10, 20, 50, 100, 200 µg/L) were used to make a standard curve.

### Cell Culture

HepG2 cells provided by China Center for Type Culture Collection (CCTCC) were grown in DMEM high glucose medium supplemented with 10% (v/v) fetal bovine serum (FBS), 1% (v/v) non-essential amino acids, 100 µg/mL penicillin and 100 µg/ml streptomycin (all from Invitrogen, Carlsbad, CA, USA). Cells were cultured at 37°C in a 5% CO_2_ incubator. For routine subculture, the cells were detached by trypsinization and subsequently split at a suitable ratio.

### Cell Viability Assay

Cell counting kit-8 (CCK-8) assay was used for the cell viability and proliferation assessment. Water-soluble tetrazolium salt (WST-8) is reduced by dehydrogenase in cells to generate a soluble yellow-color formazan dye, which is proportional to the number of living cells. The detection sensitivity of CCK-8 is higher than other tetrazolium salts like Thiazolyl Blue Tetrazolium Bromide (MTT).

HepG2 cells were seeded into 96-well microplates with a proper amount of cells per well (5000 cells in 100 µL medium for 24 hours exposure experiments and 3000 cells in 100 µL medium for 72 hours exposure experiments) and incubated overnight to adhere. Next, 10 nm AgNPs, 100 nm AgNPs or Ag^+^ (0, 1.0, 2.0, 4.0 mg/L, i.e., 0, 0.31, 0.62, 1.25 µg/cm^2^ surface area) were added to each culture well respectively, with negative controls treated with DPBS. After 12 hours to 72 hours exposure, 10 µL CCK-8 (Beyotime Institute of Biotechnology, Haimen, Jiangsu, China) was added to each well, and the plates were incubated for 1 hour at 37°C. Finally, the absorbance was measured at 450 nm using a microplate reader (Bio-rad, California, USA).

### Measurement of Reactive Oxygen Species

Intracellular ROS levels were measured by the DCF assay. HepG2 cells (1×10^6^ cells in 2.5 mL medium per well) were seeded into 6-well microplates. Next, 10 nm AgNPs (2.0 mg/L, i.e., 0.52 µg/cm^2^ surface area), 100 nm AgNPs (2.0 mg/L, i.e., 0.52 µg/cm^2^) or Ag^+^ (2.0 mg/L and 4.0 mg/L, i.e., 0.52 µg/cm^2^ and 1.04 µg/cm^2^) were added to each culture well for 24 hours, with negative controls treated with DPBS. At the end of the treatment, the cells were incubated with 5 µM 2′, 7′-dichlorodihydrofluorescein diacetate (DCFH-DA, Beyotime, China) for 30 minutes. The cells were collected, and the fluorescence of DCF, which is the oxidized product of DCFH, was analyzed immediately using a Beckman Coulter FC500 flow cytometer equipped with a 488 nm argon laser and a band pass filter of 525 nm.

### Western Blot Analysis

HepG2 cells (1×10^6^ cells in 2.5 mL medium per well) were seeded into 6-well microplates to adhere, and then exposed to a series of concentrations of 10 nm AgNPs, 100 nm AgNPs (0, 0.2, 0.5, 1.0, 2.0 mg/L, i.e., 0, 0.052, 0.13, 0.26, 0.52 µg/cm^2^ surface area) or Ag^+^ (0, 0.2, 0.5, 1.0, 2.0, 4.0 mg/L, i.e., 0, 0.052, 0.13, 0.26, 0.52, 1.04 µg/cm^2^) for 24 hours. The cells were collected in RIPA buffer supplemented with a protease inhibitor cocktail and a phosphatase inhibitor cocktail (all from Sigma, USA). After centrifuging at 12,000×g for 20 min at 4°C, the supernatant was collected and stored at −80°C for further experiments.

Each sample containing 50 µg proteins was separated by SDS-PAGE and transferred to PVDF membranes (Millipore, Bedford, MA, USA). The membranes were first blocked with 5% BSA in TBST buffer for 1 hour at room temperature and then incubated with primary antibody (1∶1000 dilution) overnight at 4°C. The membranes were then washed and incubated with HRP-conjugated secondary antibody for 2 hours. The blots were washed again, and developed using SuperSignal West Pico Chemiluminescent Substrate (Pierce, Rockford, IL, USA) according to the manufacturer’s instructions. Band intensity was analyzed by ImageJ.

### Quantitative PCR

HepG2 cells (1×10^6^ cells in 2.5 mL medium per well) were seeded into 6-well microplates to adhere, and then treated with a series of concentrations of 10 nm AgNPs or 100 nm AgNPs (0, 0.2, 0.5, 1.0, 2.0 mg/L, i.e., 0, 0.052, 0.13, 0.26, 0.52 µg/cm^2^ surface area) for 24 hours. Following chemical exposure, total RNA was extracted from the individual samples using the RNeasy mini kit (Qiagen, Hilden, Germany). The concentration of total RNA was determined at 260 nm using a Bio-Rad SmartSpec 3000 spectrophotometer (Bio-rad, California, USA). The first strand cDNA was synthesized from 1 µg of total RNA mixed with random 6-mers using the PrimeScript II 1st Strand cDNA Synthesis Kit (Takara, Dalian, China).

The expression levels of target genes were detected by real-time quantitative PCR, with housekeeping gene β-actin used as an internal control. The reactions were performed with 7300 real-time PCR system (Applied Biosystems Inc., USA) using Power SYBR Green PCR Master Mix (Applied Biosystems Inc., USA). After PCR, a melting curve analysis was performed to demonstrate the specificity of the PCR products, which displayed as a single peak. The relative expression levels of the target genes were expressed by calculating the equation 2^−ΔΔCt^, where C_t_ (cycle threshold) represents the cycle at which the fluorescence signal first significantly increases above background and ΔΔC_t_ represents (Ct_target_–Ct_actin_)_treatment_–(Ct_target_–Ct_actin_)_control_ [35]. The gene names, accession numbers in GenBank, specific primer sequences are listed in Table 1.

**Table 1:** Sequences of primers used for real-time quantitative PCR.

Target Gene	GenBank Accession No.	Product Length (bp)	Primer Sequences
c-Fos	NM_005252.3	82	Forward: 5′-CAAGCCCTCAGTGGAACC-3′
			Reverse: 5′-TGCTGGGAACAGGAAGTCAT-3′
c-Jun	NM_002228.3	87	Forward: 5′-CTCGGACCTCCTCACCTCG-3′
			Reverse: 5′-CCGTTGCTGGACTGGATTAT-3′
β-actin	NM_001101.3	149	Forward: 5′-TGCCCATCTACGAGGGGTAT-3′
			Reverse: 5′-AATGTCACGCACGATTTCC-3′

### Statistical Analysis

All data were expressed as the mean ± standard deviation (S.D.). The differences between the control and the selected treatment group were calculated by unpaired two tailed Student’s t-tests (for the CCK-8 assay and the densitometric analysis of the western blot) or paired two tailed Student’s t-tests (for the quantitative PCR). A *p*-value less than 0.05 was considered statistically significant.

## Results

### Characterization of AgNPs

**Figure 1:**
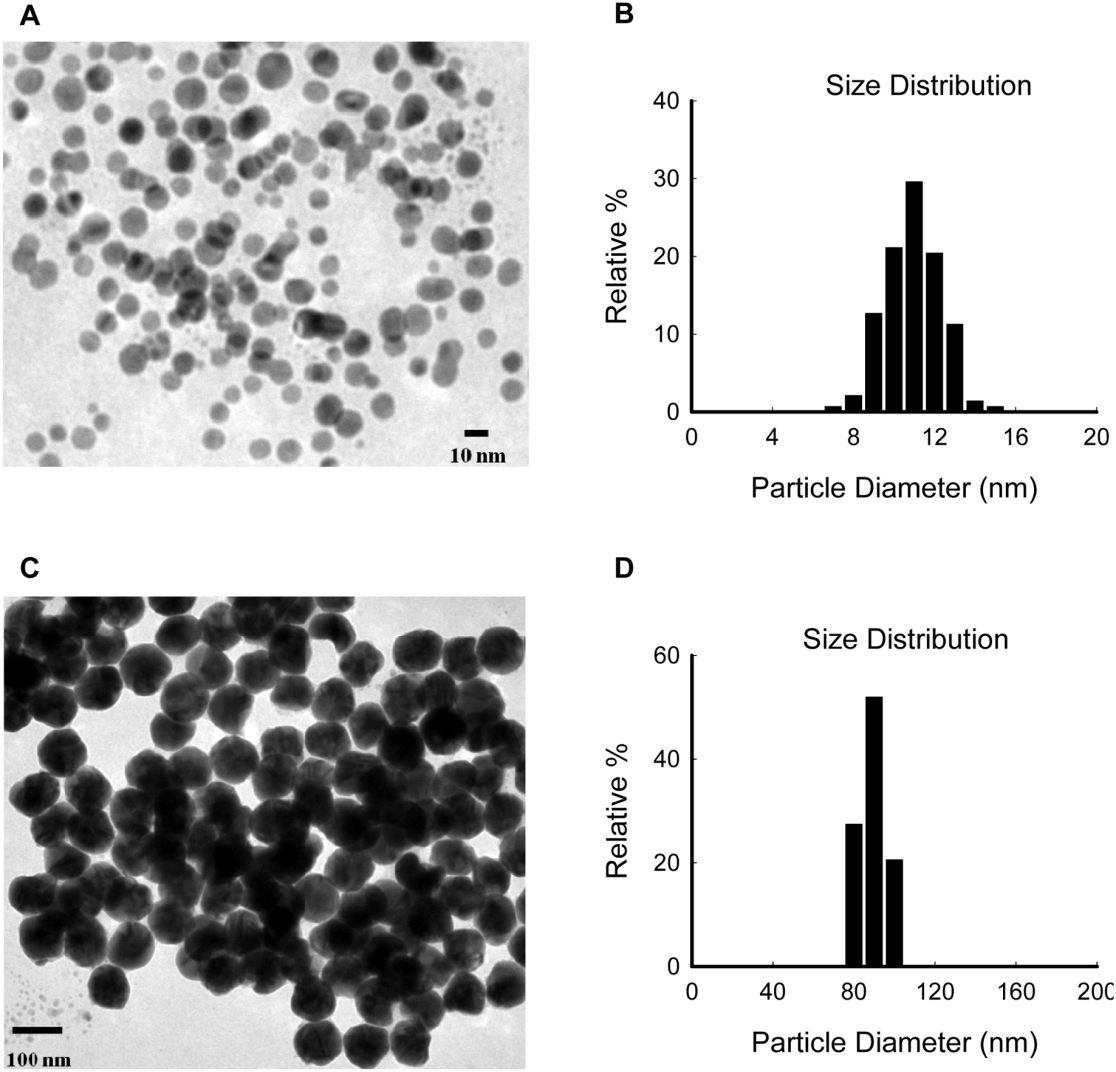
Characterization of AgNPs by TEM. (A) TEM images of 10 nm AgNPs. (B) Size distribution of 10 nm AgNPs calculated based on the TEM images. (C) TEM images of 100 nm AgNPs. (D) Size distribution of 100 nm AgNPs calculated based on the TEM images.

Two types of AgNPs dispersions purchased from Sigma were assessed in this paper. To confirm and further clarify the shapes and primary sizes of the AgNPs dispersions, TEM was used. As shown in Figure 1, the majority of the AgNPs were spherical, well-dispersed, and particle diameters were in the 8–14 nm and 75–105 nm ranges respectively. Their hydrodynamic diameters, determined by DLS, were 19.6±0.5 nm and 99.5±0.3 nm, respectively (Figure S1 and Table 2). Taking the relatively uniform size distributions into consideration (Figure 1 and S1), we still referred to them as 10 nm and 100 nm AgNPs, respectively, throughout the rest of the text.

**Table 2:** Hydrodynamic diameters of AgNPs by DLS in different conditions.

DLS Conditions		Hydrodynamic Diameters* (nm, mean ± SD, n = 3)	
		10 nm AgNPs	100 nm AgNPs
Stock solutions		19.6±0.5	99.5±0.3
1∶20 diluted in the cell culture medium	5 min	15.0±0.01	123.0±1.1
	1 h	15.1±0.04	125.1±1.3
	24 h	15.4±0.2	131.3±1.1
	48 h	17.9±1.4	144.8±0.7
	72 h	15.0±0.2	145.7±2.0

*Hydrodynamic diameter: Z-average.

Furthermore, the AgNPs diluted in the exposure cell culture medium over time (from 5 min to 72 h incubation) were characterized by DLS. The hydrodynamic diameter comparisons, relative to the stock solutions, revealed that, overall, both of the AgNPs under cellular-relevant conditions were well dispersed and did not agglomerate (Table 2). The zeta potential values were −36.2±3.7 mV and −41.1±1.0 mV for the stock solutions of 10 nm and 100 nm AgNPs, respectively. After 48 hours of incubation in the cell culture medium, they were decreased to −9.5±0.3 mV and −10.1±0.7 mV, respectively.

The total Ag contents of the AgNPs stock solutions were 20.22±0.08 mg/L and 20.13±0.59 mg/L for 10 nm and 100 nm AgNPs, respectively, in agreement with the product information. In addition, they may generate silver ions 0.62±0.02 mg/L (approximately 3.1% w/w) and 0.48±0.03 mg/L (approximately 2.4% w/w), respectively. Because of this, the low levels of Ag^+^ release would not be considered in subsequent experiments.

### Cytotoxicity of AgNPs in HepG2 Cells at Low Doses

**Figure 2:**
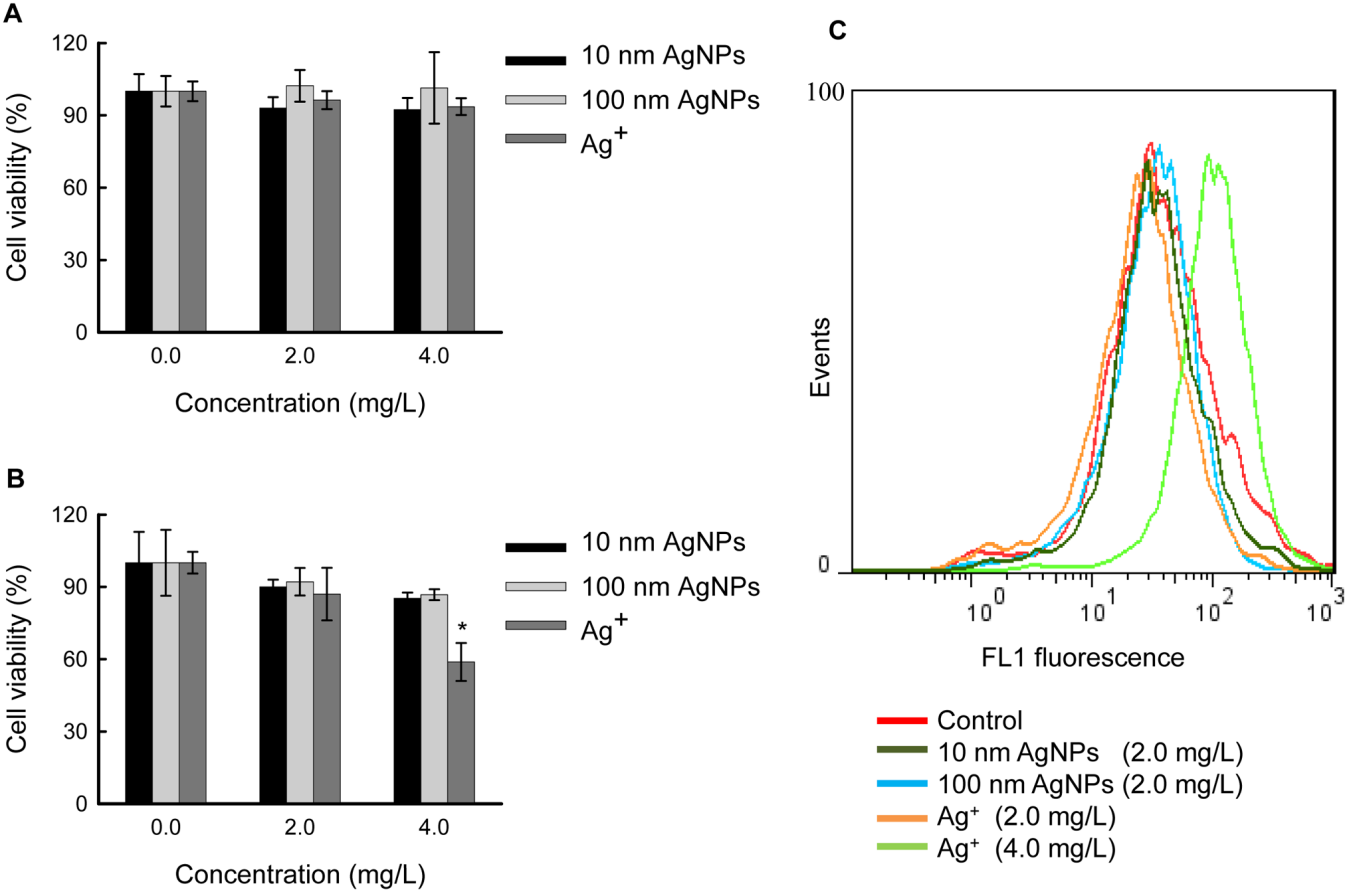
Cytotoxicity of AgNPs and Ag^+^ in HepG2 cells. (A) Cells were exposed to different concentrations of 10 nm AgNPs, 100 nm AgNPs or Ag^+^ for 12 hours, and cell viabilities were determined by the CCK-8 assay. (B) Cells were exposed to different concentrations of 10 nm AgNPs, 100 nm AgNPs or Ag^+^ for 24 hours, and cell viabilities were determined by the CCK-8 assay. The data are expressed as mean ± S.D. (n = 6), and asterisks (*) indicate a statistically significant difference compared to untreated controls (*p*<0.05). (C) Detection of ROS in HepG2 cells. The cells were exposed to 10 nm AgNPs (2.0 mg/L), 100 nm AgNPs (2.0 mg/L) or Ag^+^ (2.0 mg/L and 4.0 mg/L) for 24 hours, and then incubated with 5 µM DCFH-DA for 30 min. The fluorescence intensity was quantified using flow cytometry.

To define a non-cytotoxic concentration range, we tested the cytotoxicity of AgNPs at 2.0 mg/L and 4.0 mg/L doses in HepG2 cells by measuring the cell viabilities. After 12 hours (Figure 2A) and 24 hours (Figure 2B) exposure, neither 10 nm AgNPs nor 100 nm AgNPs displayed significant cytotoxicity. Taking the relatively low stock concentrations of AgNPs into consideration, we chose 2.0 mg/L and lower doses of AgNPs as non-cytotoxic AgNPs for exposure in the subsequent experiments.

In parallel, we performed cytotoxicity studies with Ag^+^ (provided by AgNO_3_) at the same experimental conditions. After 24 hours exposure, 4.0 mg/L Ag^+^ decreased cellular viability to approximately 59% relative to the non-exposed control (Figure 2B), whereas shorter exposure time (e.g., 12 hours) or lower exposure dose (e.g., 2.0 mg/L) did not exhibit significant cytotoxicity (Figure 2). Therefore, 2.0 mg/L and lower doses of Ag^+^ were chosen as non-cytotoxic Ag^+^ for comparisons with AgNPs.

Following the cytotoxicity experiments, intracellular ROS in AgNPs and Ag^+^ exposed HepG2 cells was detected. As shown in Figure 2C, no significant difference was observed in ROS formation in the cells exposed to both AgNPs and Ag^+^ (2.0 mg/L) compared to unexposed cells, while Ag^+^ exposure at 4.0 mg/L significantly increased the fluorescent signals (indicating ROS generation). This result is consistent with Figure 2A and 2B results and indicates that non-cytotoxic AgNPs and Ag^+^ may not induce oxidative stress in HepG2 cells.

### Low doses of AgNPs Accelerate Cell Proliferation

**Figure 3:**
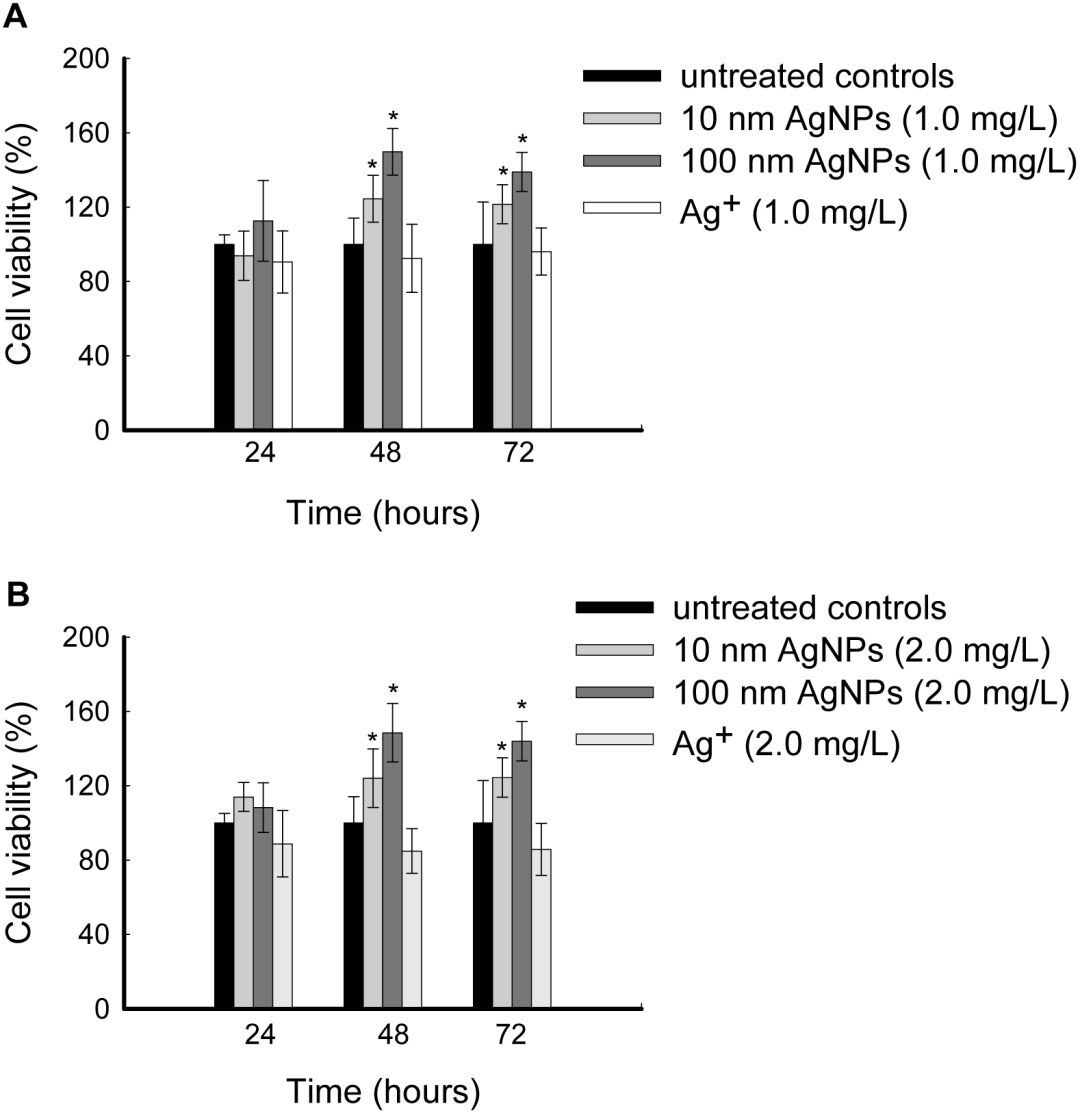
Non-cytotoxic AgNPs induced cell proliferation in HepG2 cells. (A) Cells were exposed to 1.0 mg/L of 10 nm AgNPs, 100 nm AgNPs or Ag^+^ for various time periods, and cell viabilities were determined by the CCK-8 assay. (B) Cells were exposed to 2.0 mg/L of 10 nm AgNPs, 100 nm AgNPs and Ag^+^ for various time periods and cell viabilities were determined by CCK-8 assay. The data are expressed as mean ± S.D. (n = 6), *p<0.05.

To explore the potential biological effects of non-cytotoxic AgNPs on HepG2 cells, cell growth was checked using 72 hours cell viability assays. As shown in Figure 3, after exposure to 10 nm AgNPs for 48 hours or 72 hours, the cell viabilities increased to approximately 125% relative to the unexposed controls. The increases of cell viabilities were more evident after exposure to 100 nm AgNPs, with up to approximately 150% viability relative to the unexposed controls after 48 hours exposure. These intriguing findings suggest that low doses of AgNPs accelerate cell proliferation. However, Ag^+^ at the same dose levels did not increase the cell viability.

### AgNPs at Non-cytotoxic Doses Activate p38

**Figure 4:**
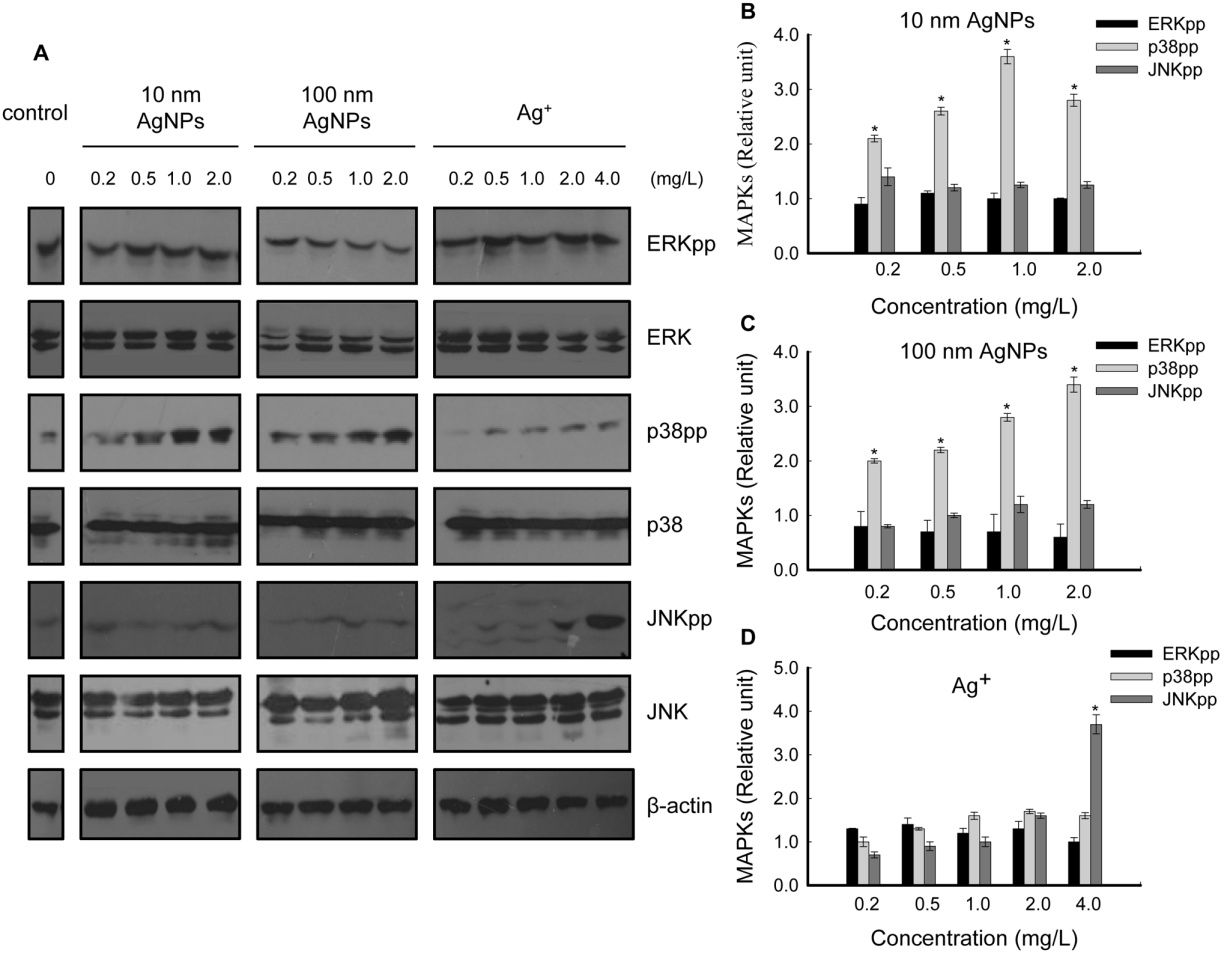
Analysis of regulation of MAPKs in HepG2 cells treated with non-cytotoxic AgNPs and Ag^+^. (A) Cells were treated with a series of concentrations of 10 nm AgNPs, 100 nm AgNPs or Ag^+^ for 24 hours, and the samples were analyzed with dual-phospho-ERK (Thr202/Tyr204) antibody, ERK antibody, dual-phospho-p38 (Thr180/Tyr182) antibody, p38 antibody, dual-phospho-JNK (Thr183/Tyr185) antibody and JNK antibody, respectively, using western blot. β-actin was used for equal loading. The panel shows images representative of three independent experiments. (B, C, D) The intensity of each chemiluminescence protein band was analyzed by ImageJ and normalized to the MAPKpp/β-actin ratio (control = 1), with the data expressed as mean ± S.D. (n = 3), *p<0.05.

The mechanisms by which AgNPs influence the proliferation of HepG2 cells remain to be investigated. Stress related MAPK signaling pathways were assessed. It is well known that MAPK family members (mainly including ERK, p38 and JNK) are the key regulators in each MAPK cascade [36], so their expression levels were examined first. HepG2 cells were treated with a series of concentrations of 10 nm AgNPs, 100 nm AgNPs or Ag^+^ for 24 hours, the samples were analyzed by western blot and the results showed that the expression of ERK, p38 and JNK were all unchanged (Figure 4A).

As the dual-phosphorylation state of each MAPK member represents the activity of corresponding MAPK cascade [36], the activation states of MAPKs induced by non-cytotoxic AgNPs were further examined. Our results demonstrated that dual-phosphorylation of p38 (Thr180/Tyr182) was significantly increased by both types of AgNPs in a concentration-dependent manner, whereas dual-phosphorylation of ERK (Thr202/Tyr204) and JNK (Thr183/Tyr185) were unaffected by AgNPs treatment (Figure 4A, B, C).

The effects of Ag^+^ were also examined. In contrast to the changes induced by AgNPs, p38 activation was not detected in Ag^+^ treated cells, while JNK was weakly activated at 2.0 mg/L (non-cytotoxic) and significantly activated at 4.0 mg/L (modestly cytotoxic) (Figure 4A, D).

### Non-cytotoxic AgNP-Induced p38 Activation Is ROS Independent

Several studies have shown the correlation between ROS generation and MAPK activation [25], [27], thus, whether intracellular ROS is required for non-cytotoxic AgNP-induced p38 activation was investigated. It has been reported that N-acetyl cysteine (NAC) is an important ROS inhibitor for its function as a precursor for glutathione synthesis [25]. In the ROS measurements, pretreatment with 10 mM NAC for 2 hours prior to 24 hours exposure with 4.0 mg/L Ag^+^ in HepG2 cells could effectively inhibit ROS generation (Figure S2), so it could be used in the subsequent assays to exclude ROS generation completely.

**Figure 5:**
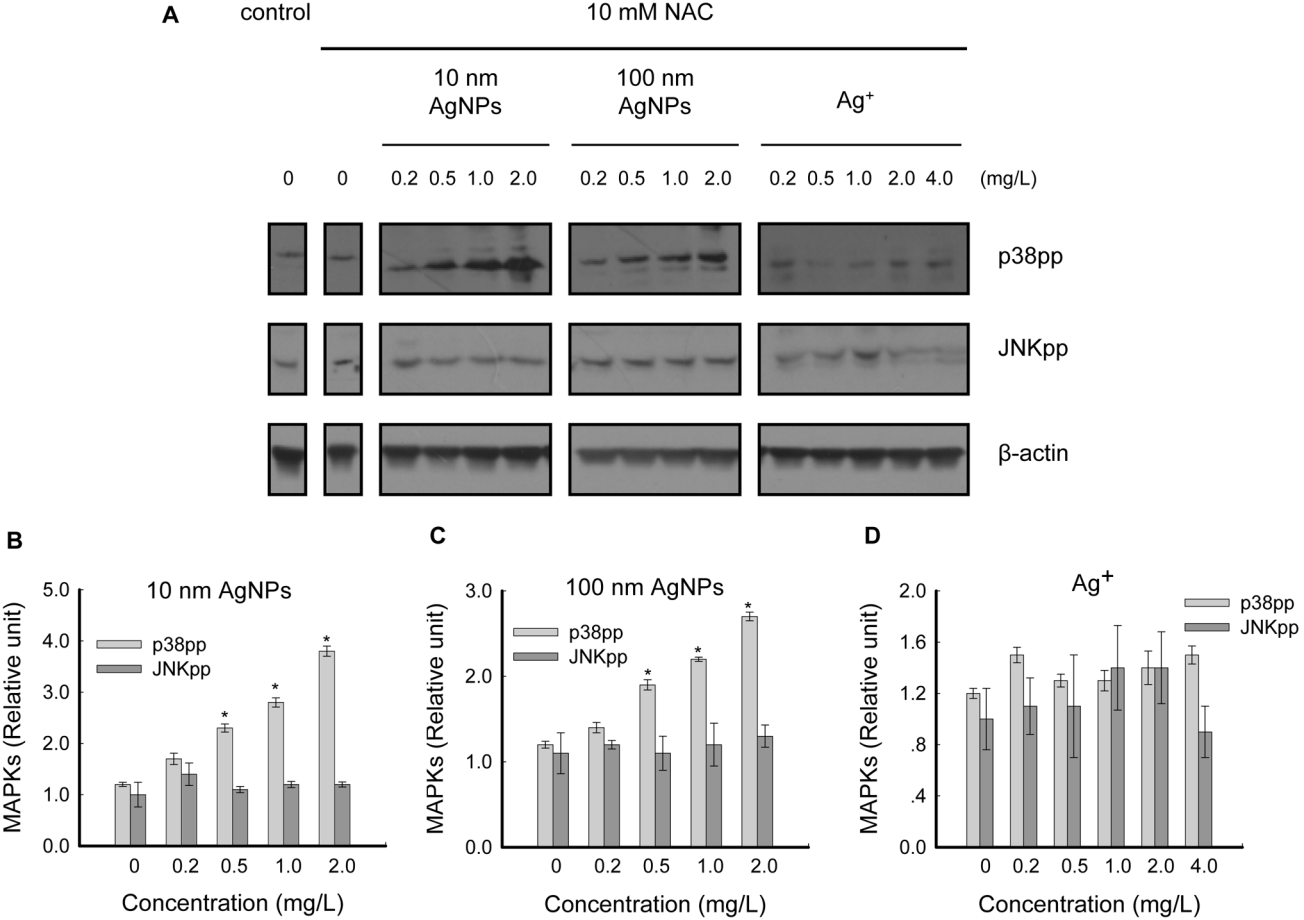
Effects of NAC on non-cytotoxic AgNP-induced p38 activation in HepG2 cells. (A) Cells were pretreated with 10 mM NAC for 2 hours prior to 24 hours exposure with a series of concentrations of 10 nm AgNPs, 100 nm AgNPs or Ag^+^, and the samples were analyzed with dual-phospho-p38 (Thr180/Tyr182) antibody and dual-phospho-JNK (Thr183/Tyr185) antibody, respectively, using western blot. β-actin was used for equal loading. The panel shows images representative of three independent experiments. (B, C, D) The intensity of each chemiluminescence protein band was analyzed by ImageJ and normalized to the MAPKpp/β-actin ratio (control = 1), with the data expressed as mean ± S.D. (n = 3), *p<0.05.

HepG2 cells were pretreated with 10 mM NAC for 2 hours prior to 24 hours exposure to a series of concentrations of 10 nm AgNPs, 100 nm AgNPs or Ag^+^. Western blot analysis showed that phosphorylation of p38 was not inhibited by NAC treatment (Figure 5A, B, C), and the trends towards increases in p38 phosphorylation were consistent with the NAC-untreated groups (Figure 4B, C, Figure 5B, C), indicating that non-cytotoxic AgNP-induced p38 activation was independent of ROS in HepG2 cells. In contrast, the phosphorylation of JNK was effectively inhibited by NAC treatment (Figure 5A, D), which means that JNK activation by Ag^+^ was mediated by ROS generation. These results were consistent with the ROS detection results in Figure 2C.

### Expression Levels of p38 Regulated Genes

**Figure 6:**
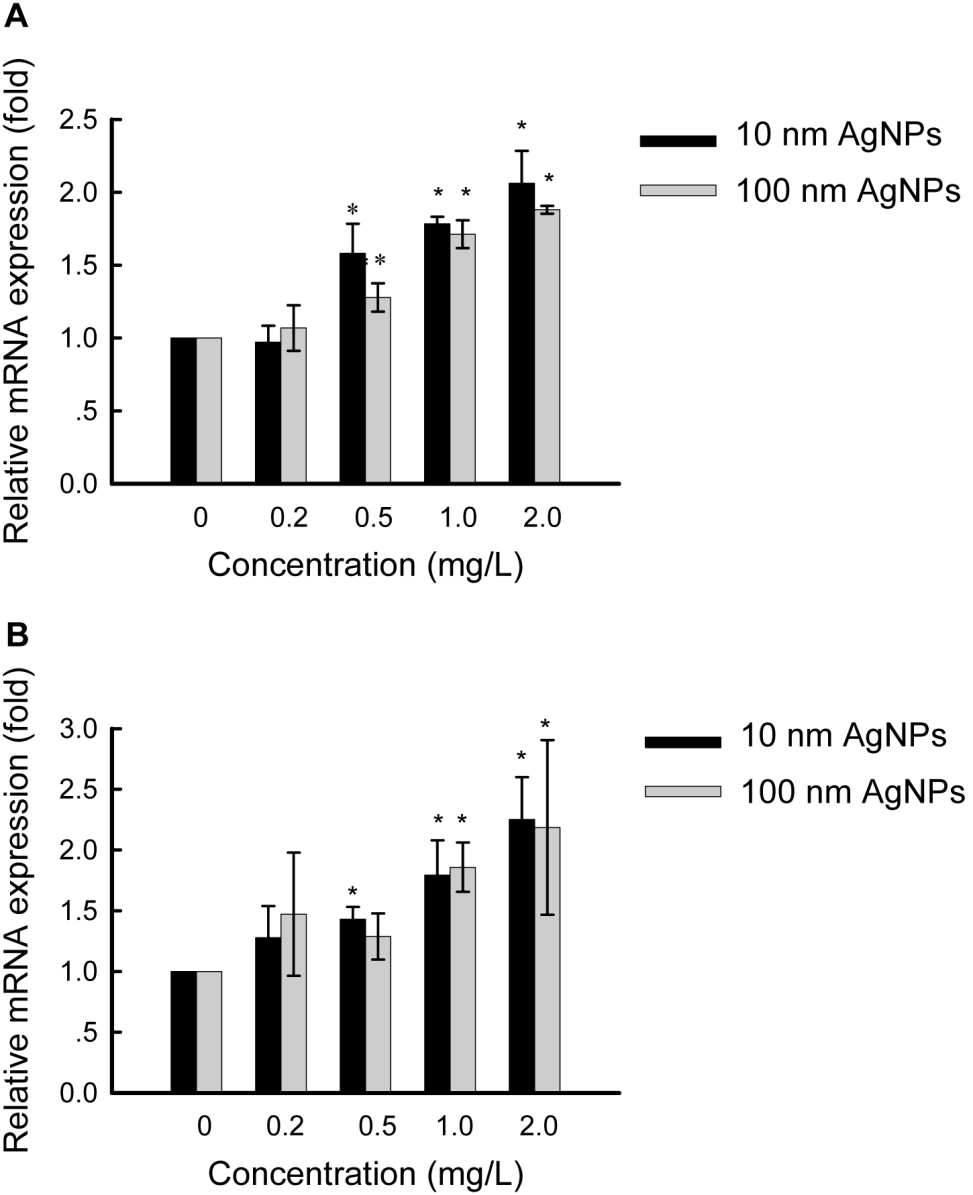
Quantitative PCR analysis of specific mRNAs in HepG2 cells treated with AgNPs. Cells were treated with various concentrations of 10-Fos (panel A) and c-Jun (panel B) were determined relative to β-actin mRNA. Values are given as mean ± S.D. (n = 3), *p<0.05.

To further analyze p38 activation induced by AgNPs at two sizes, the expression levels of p38 pathway downstream target genes c-Fos and c-Jun were studied in HepG2 cells treated for 24 hours with a series of concentrations of 10 nm AgNPs or 100 nm AgNPs. As expected, mRNA levels of both c-Fos (Figure 6A) and c-Jun (Figure 6B) were increased in a concentration-dependent manner, corresponding to p38 activation. With 10 nm and 100 nm AgNPs at 2.0 mg/L, the expression levels of c-Fos mRNA were increased 2.1- and 1.9-fold, respectively (Figure 6A), while c-Jun mRNA were increased 2.3- and 2.2-fold, respectively (Figure 6B).

### P38 Activation Is Required for Non-cytotoxic AgNP- Induced Cell Proliferation

**Figure 7:**
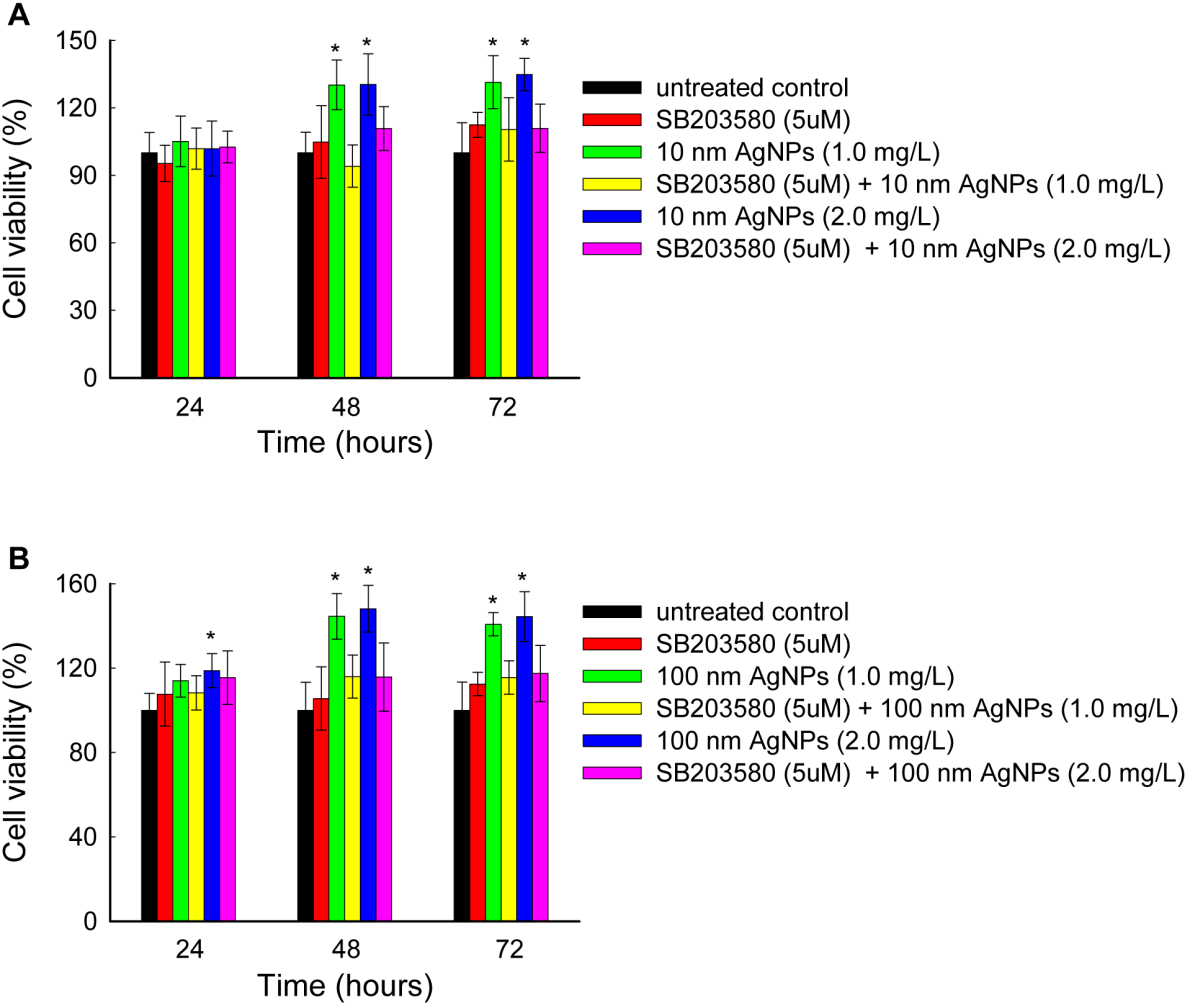
Prevention of non-cytotoxic AgNP-induced cell proliferation by p38 inhibitor pretreatment in HepG2 cells. (A) Cells were pretreated with 0 or 5 µM SB203580 for 2 hours, prior to exposure with 10 nm AgNPs (1.0 mg/L and 2.0 mg/L) for various time periods, and cell viabilities were determined by the CCK-8 assay. (B) Cells were pretreated with 0 or 5 µM SB203580 for 2 hours, prior to exposure with 100 nm AgNPs (1.0 mg/L and 2.0 mg/L) for various time periods, and cell viabilities were determined by the CCK-8 assay. The data are expressed as mean ± S.D. (n = 6), *p<0.05.

To determine whether p38 activation is required for AgNP-induced cell proliferation at low doses, we inhibited p38 activation by pre-treating cells with SB203580 [37], [38], a highly specific p38 inhibitor (Figure S3), for 2 hours, prior to exposure with 10 nm AgNPs or 100 nm AgNPs for 24 to 72 hours. As shown in Figure 7, SB203580 effectively inhibited AgNP-induced cell proliferation. For example, 10 nm AgNPs at 1.0 mg/L increased the cell viabilities to approximately 130% after 48 hours exposure, which was reduced to 94% by pretreatment with SB203580; 100 nm AgNPs at 1.0 mg/L increased the cell viabilities to approximately 145% after 48 hours exposure, which was reduced to 116% by pretreatment with SB203580. These data indicate that the stimulation effects of AgNPs are dependent on the activation of p38 in HepG2 cells.

## Discussion

In this paper, TEM combined with DLS techniques were used to characterize two types of AgNPs. The particle size analysis revealed that both of the AgNPs displayed relatively uniform size distributions. As there is an obvious difference between the sizes of the two AgNPs, they could be utilized in our study to explore the influences of size on AgNP-induced biological effects.

To confirm the behaviors of AgNPs under the experimental conditions, DLS measurements were performed under the cellular assay-relevant conditions. The hydrodynamic diameter data revealed that the AgNPs were well dispersed in the exposure cell culture medium (Table 2). However, the corresponding zeta potential were decreased relative to the stock solutions, indicating the less stability of AgNPs in the DMEM medium (with 10% FBS). The decreased zeta potential in the cell culture medium have also been reported by the existing work [22], [39]. Our results are consistent with the previous studies that FBS and citrate coating may prevent agglomeration of AgNPs [22], [40], [41].

To date, a large number of *in vitro* studies have reported that AgNPs at relatively high doses produced toxicity targeted liver and other organs mainly based on the assessment of cell viability, ROS generation, mitochondrial function and apoptosis [11]. However, reports on the biological effects induced by AgNPs at non-cytotoxic doses are limited. Kawata et al. performed DNA microarray analysis to demonstrate that non-cytotoxic AgNPs exposure altered genes involved in cell division, proliferation and DNA repair in HepG2 cells [29], thus, the aim of this study was to further evaluate potential biological effects of non-cytotoxic AgNPs at the cellular and protein levels. For this purpose, we performed the cell proliferation assays for HepG2 cells under non-cytotoxic exposure conditions of AgNPs (100% cell viability detected by the cell cytotoxicity assay). Contrast to the high doses of AgNPs induced cytotoxicity, our results showed that low doses of AgNPs accelerated cell proliferation significantly (Figure 3). This phenomenon has rarely been reported [42] and may be explained as “hormesis”, which is characterized by a low dose stimulation by potentially toxic agents [43], [44].

In fact, the hormesis effects could also be observed in the previous studies. Shin et al. studied the cytotoxicity of AgNPs on peripheral blood mononuclear cells, and the results showed low dose stimulation and high dose inhibition of cell proliferation [45]. Arora et al. studied the cellular responses to AgNPs in the human skin carcinoma cell line A431 and the human fibrosarcoma cell line HT-1080. A particularly interesting thing was the hormesis effects of AgNPs on caspase-3 activity [46]. Our results and these examples gathered from the literature led us to suggest that AgNP-induced hormesis effects are ubiquitous to the organisms. However, these papers focused on the high dose inhibition and did not give a further explanation for the low dose stimulation.

To explore the molecular mechanisms of non-cytotoxic AgNP-induced cell proliferation, the regulation of MAPKs were assessed for two reasons: MAPK cascades are involved in the regulation of cell proliferation in many physiological activities [47], [48], and up-regulation of MAPKs have been shown to occur in response to treatment with high doses of AgNPs in both mammalian cells [27], [28] and non-mammalian animal models [49], [50]. The present study revealed increased expressions of p38pp, c-Jun and c-Fos on exposure to non-cytotoxic AgNPs (Figure 4, 6, 7), which strongly suggests that the p38 MAPK pathway, via c-Jun and c-Fos, is involved in the stimulation effects of AgNPs in HepG2 cells. It has been demonstrated that c-Jun and c-Fos, components of AP-1 (activator protein-1, an important transcription factor), may promote proliferation in several cell types [51]. We propose that upon stimulation by non-cytotoxic AgNPs, the p38 pathway may induce AP-1 activity by participating in c-Jun and c-Fos up-expression. AP-1, in turn, mediates the regulation of cell cycle-related genes to control cell proliferation [29], [52]. This may protect cells from damage.

It is worth noting that p38 activation has also been reported to be involved in cytotoxicity induced by high doses of AgNPs [28], which indicates that p38 may exhibit dual regulation in response to exposure to AgNPs: at low dose levels, p38 initiates a protective response; but with increasing AgNPs concentration, p38 transitions to a damaging response. However, as the existing studies used different types of AgNPs and different exposure models, more systematic experiments are needed to elucidate the roles of p38 in AgNPs exposure.

Size has been regarded as a major influencing factor in AgNP-induced toxicity [31], [53], [54], [55], [56]. Generally, smaller NPs possess larger specific surface areas to facilitate higher reactivity [57]. However, according to our results, 10 nm and 100 nm AgNPs at the same mass did not exhibit dramatic differences in cell proliferation or p38 activation (Figure 3, 4). Additionally, p38 downstream c-Fos and c-Jun mRNA were increased to the same degree by both sizes of AgNPs (Figure 6), indicating slightly size dependent AgNP-induced hormesis. It is still unclear why HepG2 cells showed such a similar sensitivity to different sizes of AgNPs. Amro M. EL Badawy et al. also found that particle shape and size had minimal influence on the toxicity of the evaluated AgNPs, while surface charge was a dominant factor in determining their toxicity [58]. Thus, other factors, including cell type and surface modification (or surface charge) of AgNPs, will also need to be considered to evaluate the hormesis effects of AgNPs.

According to our results, the cytotoxicity of Ag^+^ correlates with ROS generation (oxidative stress) (consistent with [25]) and JNK MAPK activation (Figure 2, 4, 5). However, within its non-cytotoxic concentration range, the promotion of cell proliferation was not observed, corresponding to the unchanged states of p38 (Figure 4). Therefore, the present study suggests that HepG2 cells display different susceptibilities to AgNPs and Ag^+^ under non-cytotoxic conditions. Similarly, Hyun-Jeong et al. also reported the selective toxicity of AgNPs, but not Ag^+^, on Jurkat T cells through p38 activation [28].

For a long time, whether AgNPs exert particle-specific toxicity remains ambiguous [59]. Eun-Jung Park et al. reported that AgNPs were ionized in the cells to cause cytotoxicity by a Trojan-horse type mechanism in RAW264.7 cells [40]. Xiu et al. also reported that AgNPs displayed antimicrobial activity, primarily relying on Ag^+^ release, with negligible particle-specific antibacterial activity [60]. Alternatively, some investigators suggested that both AgNPs and Ag^+^ may be responsible for the toxicity of AgNPs [29]. In contrast, Kim et al. suggested that AgNPs induced toxicity independent of Ag^+^ in HepG2 cells [25]. Amro M. EL Badawy et al. demonstrated that the mechanisms of AgNPs toxicity may involve a combination of both physical and chemical interactions, different from the toxicity effect caused by Ag^+^ [58]. In our study, the different regulation patterns between AgNPs and Ag^+^ inferred that the hormesis effect in HepG2 cells induced by AgNPs is an intrinsic effect of AgNPs independent of free Ag^+^.

To conclude, our results demonstrated the hormesis effects of AgNPs under non-cytotoxic conditions *in vitro* and elucidated the behind molecular mechanisms for the first time. Two representative AgNPs with different particle sizes at non-cytotoxic doses induced p38 MAPK pathway activation and led to the promotion of HepG2 cell proliferation. More studies are being conducted to explore other relevant regulatory factors in primary hepatocytes, which could more closely resemble the tissue environment relative to HepG2 cells [21]. Although hormesis is considered an adaptive response, subsequent evaluation of the biological and ecological context of this response should be considered [61]. More importantly, due to the complexity of the interaction between AgNPs and living organisms, the potential long-term effects of AgNPs at low doses on human beings should be further assessed to set a foundation for their rational applications.

## Supporting Information

Figure S1 **DLS size distributions of AgNPs stock solutions by intensity.** The black line with diamond dots and the red line with circle dots represent the hydrodynamic diameter distributions of 10 nm AgNPs and 100 nm AgNPs, respectively.
(DOC)

Figure S2 **The function of NAC on inhibiting ROS generation.** The cells were pretreated with 10 mM NAC for 2 hours prior to 24 hours exposure with 4.0 mg/L Ag^+^ in HepG2 cells. After incubation with 5 µM DCFH-DA for 30 min, the fluorescence intensity was quantified using flow cytometry.
(DOC)

Figure S3 **The function of SB203580 on inhibiting p38pp formation.** Cells were pretreated with 0 and 5 µM SB203580 for 2 hours, prior to exposure with 1.0 mg/L of 10 nm AgNPs or 100 nm AgNPs for 24 hours. The samples were analyzed with dual-phospho-p38 (Thr180/Tyr182) antibody using western blot. β-actin was used for equal loading.
(DOC)
