# Supplementary material for: Hormesis Effects of Silver Nanoparticles at Non-Cytotoxic Doses to Human Hepatoma Cells
Source: PLoS One. 2014 Jul 17;9(7):e102564. doi: 10.1371/journal.pone.0102564 (PMC4102499; doi:10.1371/journal.pone.0102564)

**Figure S1.** DLS size distributions of AgNPs stock solutions by intensity. The black line with diamond dots and the red line with circle dots represent the hydrodynamic diameter distributions of 10 nm AgNPs and 100 nm AgNPs, respectively.


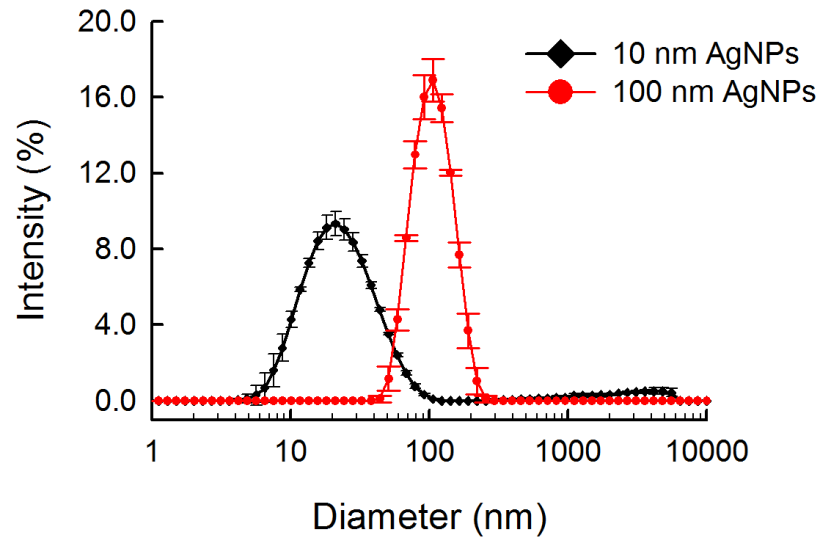

Supplement: Figure S1 — DLS size distributions of AgNPs stock solutions by intensity. The black line with diamond dots and the red line with circle dots represent the hydrodynamic diameter distributions of 10 nm AgNPs and 100 nm AgNPs, respectively. (DOC) [file pone.0102564.s001.doc]
