# Supplementary material for: Hormesis Effects of Silver Nanoparticles at Non-Cytotoxic Doses to Human Hepatoma Cells
Source: PLoS One. 2014 Jul 17;9(7):e102564. doi: 10.1371/journal.pone.0102564 (PMC4102499; doi:10.1371/journal.pone.0102564)

**Figure S2.** The function of NAC on inhibiting ROS generation. The cells were pretreated with 10 mM NAC for 2 hours prior to 24 hours exposure with 4.0 mg/L Ag^+^ in HepG2 cells. After incubation with 5μM DCFH-DA for 30 min, the fluorescence intensity was quantified using flow cytometry.


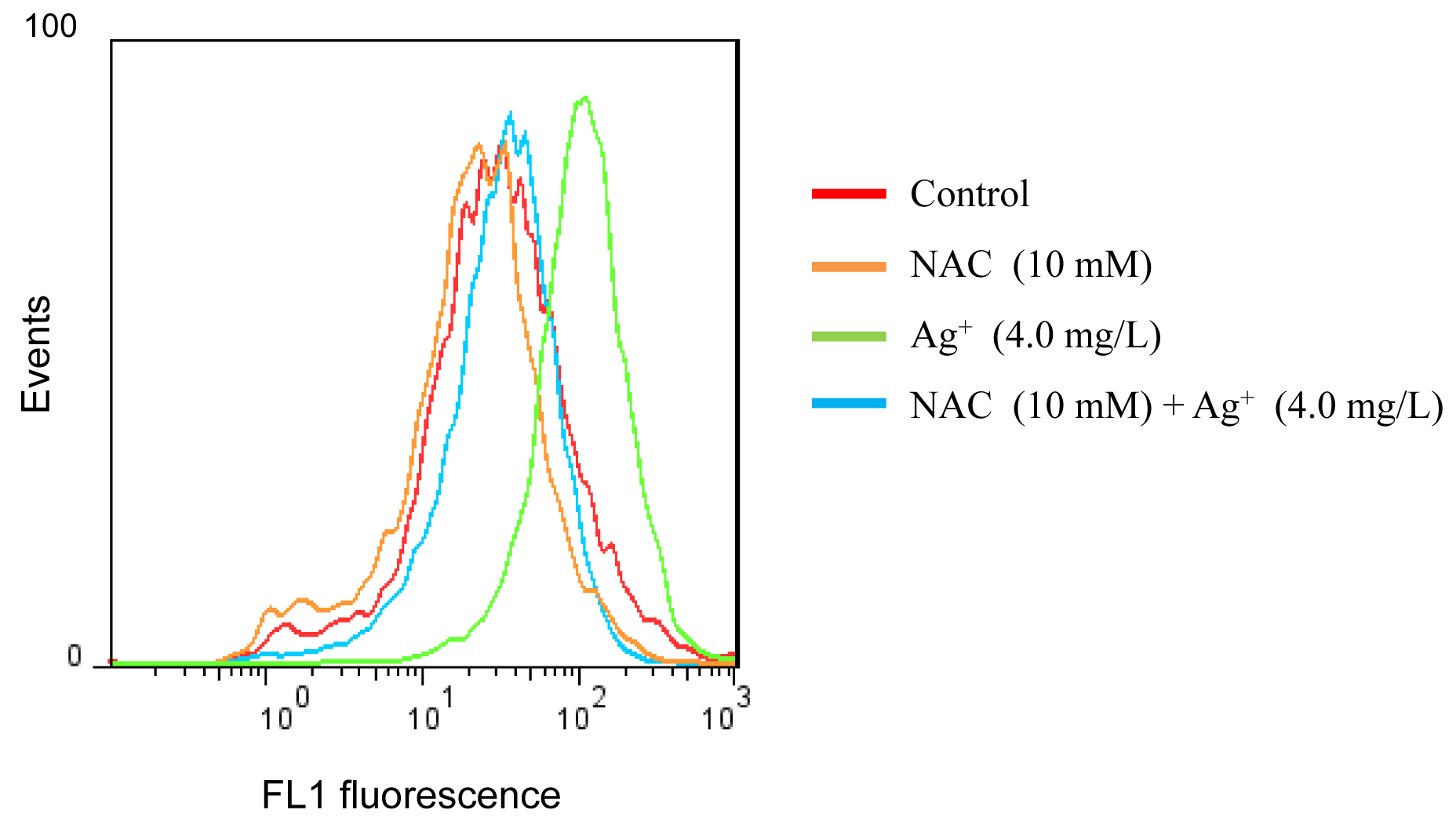

Supplement: Figure S2 — The function of NAC on inhibiting ROS generation. The cells were pretreated with 10 mM NAC for 2 hours prior to 24 hours exposure with 4.0 mg/L Ag+ in HepG2 cells. After incubation with 5 µM DCFH-DA for 30 min, the fluorescence intensity was quantified using flow cytometry. (DOC) [file pone.0102564.s002.doc]
