# Supplementary material for: Hormesis Effects of Silver Nanoparticles at Non-Cytotoxic Doses to Human Hepatoma Cells
Source: PLoS One. 2014 Jul 17;9(7):e102564. doi: 10.1371/journal.pone.0102564 (PMC4102499; doi:10.1371/journal.pone.0102564)

**Figure S3.** The function of SB203580 on inhibiting p38pp formation. Cells were pretreated with 0 and 5 μM SB203580 for 2 hours, prior to exposure with 1.0 mg/L of 10 nm AgNPs or 100 nm AgNPs for 24 hours. The samples were analyzed with dual-phospho-p38 (Thr180/Tyr182) antibody using western blot. β-actin was used for equal loading.


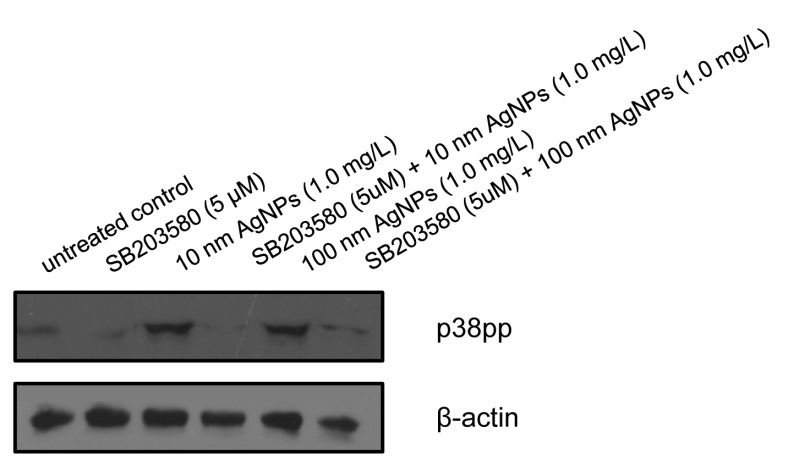

Supplement: Figure S3 — The function of SB203580 on inhibiting p38pp formation. Cells were pretreated with 0 and 5 µM SB203580 for 2 hours, prior to exposure with 1.0 mg/L of 10 nm AgNPs or 100 nm AgNPs for 24 hours. The samples were analyzed with dual-phospho-p38 (Thr180/Tyr182) antibody using western blot. β-actin was used for equal loading. (DOC) [file pone.0102564.s003.doc]
